# Dimethyl *cis*-4-hydroxy­methyl­piperidine-2,6-dicarboxyl­ate

**DOI:** 10.1107/S1600536810010925

**Published:** 2010-04-02

**Authors:** Jens Hartung, Georg Stapf, Uwe Bergsträsser

**Affiliations:** a Fachbereich Chemie, Organische Chemie, Technische Universität Kaiserslautern, Erwin-Schrödinger-Strasse, D-67663 Kaiserslautern, Germany

## Abstract

The heterocyclic core of the title compound, C_10_H_17_NO_5_, adopts a chair conformation with its three C substituents positioned equatorially. In the crystal, inter­molecular O—H⋯N hydrogen bonds between neighbouring mol­ecules lead to chains along *b*. These chains are connected by hydro­phobic inter­actions, forming infinite layers and N—H⋯O=C contacts between mol­ecules of adjacent layers give rise to a three-dimensional structure.

## Related literature

For structures of related *N*-heterocyclic compounds, see: Parkin *et al.* (2004 ▶). For the synthetic procedure, see: Tang *et al.* (2006 ▶).
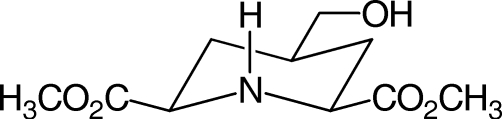


## Experimental

### 

#### Crystal data

C_10_H_17_NO_5_
*M* *_r_* = 231.25
Monoclinic, 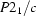

*a* = 9.1403 (4) Å
*b* = 7.9153 (3) Å
*c* = 16.0199 (6) Å
β = 90.503 (4)°
*V* = 1158.97 (8) Å^3^
*Z* = 4
Mo *K*α radiation
μ = 0.11 mm^−1^
*T* = 150 K
0.35 × 0.25 × 0.20 mm

#### Data collection

Oxford Diffraction Xcalibur diffractometer with a Sapphire CCD detector
9123 measured reflections
3542 independent reflections
1862 reflections with *I* > 2σ(*I*)
*R*_int_ = 0.045

#### Refinement

*R*[*F* ^2^ > 2σ(*F* ^2^)] = 0.044
*wR*(*F* ^2^) = 0.146
*S* = 0.89
3542 reflections
149 parameters
H atoms treated by a mixture of independent and constrained refinement
Δρ_max_ = 0.41 e Å^−3^
Δρ_min_ = −0.40 e Å^−3^

### 

Data collection: *CrysAlis CCD* (Oxford Diffraction, 2007 ▶); cell refinement: *CrysAlis CCD*; data reduction: *CrysAlis RED* (Oxford Diffraction, 2007 ▶); program(s) used to solve structure: *SHELXS97* (Sheldrick, 2008 ▶); program(s) used to refine structure: *SHELXL97* (Sheldrick, 2008 ▶); molecular graphics: *ORTEP-3* (Farrugia, 1997 ▶); software used to prepare material for publication: *WinGX* (Farrugia, 1999 ▶).

## Supplementary Material

Crystal structure: contains datablocks global, I. DOI: 10.1107/S1600536810010925/im2176sup1.cif

Structure factors: contains datablocks I. DOI: 10.1107/S1600536810010925/im2176Isup2.hkl

Additional supplementary materials:  crystallographic information; 3D view; checkCIF report

## Figures and Tables

**Table 1:** Hydrogen-bond geometry (Å, °)

*D*—H⋯*A*	*D*—H	H⋯*A*	*D*⋯*A*	*D*—H⋯*A*
O91—H91⋯N1^i^	0.82	2.07	2.883 (2)	171
N1—H1⋯O4^ii^	0.98 (2)	2.19 (2)	3.144 (2)	165 (2)

Symmetry codes: (i) 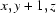; (ii) 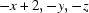.

**Fig. 1.**
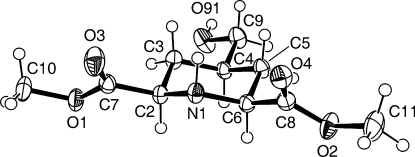
View of (I) (50% probability displacement ellipsoids)

**Fig. 2.**
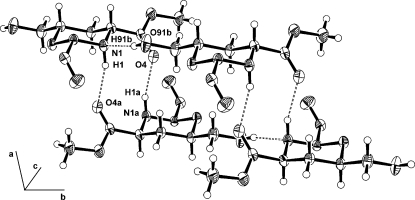
Three dimensional structure of (I) in the solid state with intermolecular O–H···N bridges and N–H···O═C contacts (50% probability displacement ellipsoids for all non H-atoms).

## supplementary crystallographic information

### Comment

The all-*cis*-isomer of 4-(hydroxymethyl)-piperidine-2,6-dicarboxylic acid dimethyl ester, (I), represents a potential tridentate ONO-donor ligand for development of a new generation of solid phase-bound oxidation catalysts. Its three C substituents were equatorially attached to piperidine adopting a ^1^C_4_-chair conformation (Figure 1). A quartett structure (*J* = 12.4 Hz) in the nuclear magnetic resonance (NMR) spectrum for axially connected protons to C3 and C5 provided evidence that this arrangement corresponded to the most significantly populated conformer of (I) in solution (CDCl_3_, 298 K). The amino H-atom was found in axial position. The arrangement that is thermodynamically favored, i.e. equatorial NH positioning, was reported for the crystal structure of piperidine and its structurally closely related derivatives morpholine and piperazine (Parkin *et al.*, 2004).

Intermolecular O–H···N bridges between proximate molecules lead to chains along *b*. The chains are additionally connected by hydrophobic interactions to form layers. N–H···O═C contacts between molecules of adjascent layers give rise to a three dimensional structure (Figure 2).

### Experimental

Pd/C [200 mg, 10 % (w/w)] was added to a solution of dimethyl 4-(hydroxymethyl)-pyridine-2,5-dicarboxylate (400 mg, 1.78 mmol; Tang *et al.*, 2006) in AcOEt (100 ml). The mixture was transferred into a hydrogenating apparatus and stirred at a pressure of 3.5 bar for 14 h at 298 K in an atmosphere of H_2_. The hydrogen pressure constantly decrased until a constant value of 2.4 bar was reached when the reaction stopped. The reaction mixture was filtrated through a short pad of celite. The filtrate was concentrated under reduced pressure. The remaining oil was covered with a layer of AcOEt (5 ml) and allowed to rest for 1 h at 277 K. Colorless crystals that deposited were collected by filtration. Yield: 139 mg (34 %); mp 372 K. ^1^H NMR (400 MHz, CDCl_3_, δ_H_ p.p.m.): 1.15 (*q*, *J* = 12.4 Hz, 2H), 1.78 (*m*, 1H), 2.13 (*d*, *J* = 12.4 Hz, 2H), 3.43 (*dd*, *J* = 11.7 Hz, 2.5 Hz, 2H), 3.55 (*d*, *J* = 6.3 Hz, 2H), 3.74 (*s*, 6H). ^13^C NMR (101 MHz, CDCl_3_δ_C_ p.p.m.): 31.7, 38.7, 52.1, 57.9, 67.4, 172.7. Analytical data calculated for all-cis-4-(hydroxymethyl)-piperidine-2,6-dicarboxylic acid dimethyl ester: C, 51.94; H, 7.41; N, 6.06; found: C, 52.34; H, 7.46; N, 6.02.

### Refinement

The hydrogen bonded to N1 was refined freely. The hydrogen bonded to O91 was positioned as idealized OH group with C—O—H angle tetrahedral and as riding atom with U_iso_(H)=1.5 times U_eq_(O91). All other H Atoms were positioned geometrically and treated as riding atoms (C—H = 0.97-0.98 Å), with U_iso_(H)=1.2 or 1.5 times U_eq_(C).

### Crystal data

**Table:**

C_10_H_17_NO_5_	*F*(000) = 496
*M**_r_* = 231.25	*D*_x_ = 1.325 Mg m^−^^3^
Monoclinic, *P*2_1_/*c*	Mo *K*α radiation, λ = 0.71073 Å
Hall symbol: -P 2ybc	Cell parameters from 2439 reflections
*a* = 9.1403 (4) Å	θ = 3.6–31.2°
*b* = 7.9153 (3) Å	µ = 0.11 mm^−^^1^
*c* = 16.0199 (6) Å	*T* = 150 K
β = 90.503 (4)°	Prism, colourless
*V* = 1158.97 (8) Å^3^	0.35 × 0.25 × 0.20 mm
*Z* = 4	

### Data collection

**Table:**

Oxford Diffraction Xcalibur diffractometer with a Sapphire CCD detector	1862 reflections with *I* > 2σ(*I*)
Radiation source: Enhance (Mo) X-ray Source	*R*_int_ = 0.045
graphite	θ_max_ = 31.1°, θ_min_ = 3.6°
Detector resolution: 16.1399 pixels mm^-1^	*h* = −13→13
Rotation method data acquisition using ω and phi scans	*k* = −11→11
9123 measured reflections	*l* = −23→23
3542 independent reflections	

### Refinement

**Table:**

Refinement on *F*^2^	0 restraints
Least-squares matrix: full	H atoms treated by a mixture of independent and constrained refinement
*R*[*F*^2^ > 2σ(*F*^2^)] = 0.044	*w* = 1/[σ^2^(*F*_o_^2^) + (0.0827*P*)^2^] where *P* = (*F*_o_^2^ + 2*F*_c_^2^)/3
*wR*(*F*^2^) = 0.146	(Δ/σ)_max_ < 0.001
*S* = 0.89	Δρ_max_ = 0.41 e Å^−^^3^
3542 reflections	Δρ_min_ = −0.40 e Å^−^^3^
149 parameters	

### Special details

**Table:**

Geometry. All esds (except the esd in the dihedral angle between two l.s. planes) are estimated using the full covariance matrix. The cell esds are taken into account individually in the estimation of esds in distances, angles and torsion angles; correlations between esds in cell parameters are only used when they are defined by crystal symmetry. An approximate (isotropic) treatment of cell esds is used for estimating esds involving l.s. planes.

### Fractional atomic coordinates and isotropic or equivalent isotropic displacement parameters (Å^2^)

**Table:**

	*x*	*y*	*z*	*U*_iso_*/*U*_eq_	
N1	0.74419 (16)	0.02707 (16)	0.01230 (8)	0.0237 (3)	
C2	0.64855 (17)	0.13614 (18)	0.06151 (9)	0.0220 (3)	
H2	0.5507	0.133	0.0361	0.026*	
O4	0.94904 (13)	−0.11150 (15)	−0.09219 (7)	0.0312 (3)	
C8	0.85668 (18)	−0.01946 (19)	−0.12238 (10)	0.0249 (3)	
C7	0.63717 (18)	0.0686 (2)	0.14932 (9)	0.0255 (3)	
O1	0.51869 (14)	0.13296 (16)	0.18655 (7)	0.0320 (3)	
C5	0.80958 (17)	0.27664 (19)	−0.07663 (10)	0.0241 (3)	
H5A	0.9091	0.2831	−0.0554	0.029*	
H5B	0.8093	0.3154	−0.1341	0.029*	
C4	0.71060 (17)	0.39036 (18)	−0.02456 (9)	0.0223 (3)	
H4	0.6126	0.3887	−0.0498	0.027*	
O91	0.67225 (15)	0.67327 (15)	0.02363 (8)	0.0405 (3)	
H91	0.7015	0.7712	0.0231	0.061*	
O3	0.72305 (16)	−0.02473 (18)	0.18282 (8)	0.0434 (4)	
C6	0.75608 (17)	0.09224 (19)	−0.07327 (9)	0.0226 (3)	
H6	0.6588	0.0873	−0.0994	0.027*	
C3	0.69913 (18)	0.32103 (19)	0.06401 (9)	0.0244 (3)	
H3A	0.6298	0.388	0.0954	0.029*	
H3B	0.7936	0.3284	0.0918	0.029*	
O2	0.83440 (15)	−0.00053 (17)	−0.20437 (7)	0.0370 (3)	
C9	0.7656 (2)	0.5714 (2)	−0.02568 (11)	0.0312 (4)	
H9A	0.8647	0.576	−0.0036	0.037*	
H9B	0.7667	0.6132	−0.0826	0.037*	
C10	0.4996 (2)	0.0889 (3)	0.27333 (10)	0.0396 (5)	
H10A	0.4118	0.1406	0.2937	0.059*	
H10B	0.582	0.1285	0.3053	0.059*	
H10C	0.4921	−0.0316	0.2786	0.059*	
C11	0.9277 (2)	−0.1006 (3)	−0.25831 (11)	0.0452 (5)	
H11A	0.903	−0.0782	−0.3156	0.068*	
H11B	0.9136	−0.2184	−0.2467	0.068*	
H11C	1.0282	−0.0712	−0.2482	0.068*	
H1	0.844 (2)	0.032 (2)	0.0344 (10)	0.029 (5)*	

### Atomic displacement parameters (Å^2^)

**Table:**

	*U*^11^	*U*^22^	*U*^33^	*U*^12^	*U*^13^	*U*^23^
N1	0.0317 (7)	0.0181 (6)	0.0214 (6)	−0.0003 (5)	0.0046 (5)	0.0005 (5)
C2	0.0248 (7)	0.0212 (7)	0.0200 (7)	0.0011 (6)	0.0032 (5)	0.0000 (6)
O4	0.0339 (7)	0.0266 (6)	0.0333 (6)	0.0032 (5)	0.0035 (5)	−0.0026 (5)
C8	0.0287 (8)	0.0208 (7)	0.0251 (7)	−0.0036 (6)	0.0040 (6)	−0.0041 (6)
C7	0.0321 (8)	0.0213 (7)	0.0232 (7)	−0.0057 (7)	0.0031 (6)	0.0016 (6)
O1	0.0380 (7)	0.0373 (7)	0.0207 (5)	−0.0014 (5)	0.0079 (5)	0.0013 (5)
C5	0.0274 (8)	0.0190 (7)	0.0259 (7)	−0.0013 (6)	0.0064 (6)	0.0014 (6)
C4	0.0253 (7)	0.0184 (7)	0.0231 (7)	−0.0027 (6)	0.0043 (6)	0.0002 (6)
O91	0.0442 (8)	0.0196 (5)	0.0581 (8)	0.0000 (5)	0.0194 (6)	−0.0033 (6)
O3	0.0477 (8)	0.0485 (8)	0.0339 (7)	0.0122 (7)	0.0056 (6)	0.0150 (6)
C6	0.0251 (7)	0.0217 (7)	0.0212 (7)	−0.0027 (6)	0.0033 (5)	−0.0021 (6)
C3	0.0313 (9)	0.0189 (7)	0.0230 (7)	−0.0006 (6)	0.0033 (6)	−0.0009 (6)
O2	0.0438 (7)	0.0435 (7)	0.0237 (6)	0.0123 (6)	0.0059 (5)	−0.0065 (5)
C9	0.0373 (9)	0.0213 (7)	0.0352 (9)	−0.0015 (7)	0.0100 (7)	0.0010 (7)
C10	0.0503 (11)	0.0494 (11)	0.0193 (7)	−0.0053 (9)	0.0072 (7)	0.0037 (8)
C11	0.0501 (12)	0.0546 (12)	0.0312 (9)	0.0183 (10)	0.0096 (8)	−0.0111 (9)

### Geometric parameters (Å, °)

**Table:**

N1—C2	1.4640 (19)	C4—C3	1.526 (2)
N1—C6	1.4696 (19)	C4—H4	0.98
N1—H1	0.98 (2)	O91—C9	1.420 (2)
C2—C7	1.509 (2)	O91—H91	0.82
C2—C3	1.535 (2)	C6—H6	0.98
C2—H2	0.98	C3—H3A	0.97
O4—C8	1.212 (2)	C3—H3B	0.97
C8—O2	1.3358 (19)	O2—C11	1.454 (2)
C8—C6	1.503 (2)	C9—H9A	0.97
C7—O3	1.201 (2)	C9—H9B	0.97
C7—O1	1.341 (2)	C10—H10A	0.96
O1—C10	1.4452 (18)	C10—H10B	0.96
C5—C4	1.529 (2)	C10—H10C	0.96
C5—C6	1.540 (2)	C11—H11A	0.96
C5—H5A	0.97	C11—H11B	0.96
C5—H5B	0.97	C11—H11C	0.96
C4—C9	1.519 (2)		
			
C2—N1—C6	110.16 (12)	N1—C6—C5	113.04 (12)
C2—N1—H1	110.1 (10)	C8—C6—C5	110.08 (12)
C6—N1—H1	104.3 (10)	N1—C6—H6	108.1
N1—C2—C7	109.83 (12)	C8—C6—H6	108.1
N1—C2—C3	113.27 (12)	C5—C6—H6	108.1
C7—C2—C3	109.67 (12)	C4—C3—C2	109.98 (12)
N1—C2—H2	108	C4—C3—H3A	109.7
C7—C2—H2	108	C2—C3—H3A	109.7
C3—C2—H2	108	C4—C3—H3B	109.7
O4—C8—O2	124.00 (14)	C2—C3—H3B	109.7
O4—C8—C6	124.87 (14)	H3A—C3—H3B	108.2
O2—C8—C6	111.10 (14)	C8—O2—C11	115.98 (14)
O3—C7—O1	124.19 (14)	O91—C9—C4	109.19 (13)
O3—C7—C2	125.75 (15)	O91—C9—H9A	109.8
O1—C7—C2	110.01 (13)	C4—C9—H9A	109.8
C7—O1—C10	116.17 (14)	O91—C9—H9B	109.8
C4—C5—C6	110.44 (12)	C4—C9—H9B	109.8
C4—C5—H5A	109.6	H9A—C9—H9B	108.3
C6—C5—H5A	109.6	O1—C10—H10A	109.5
C4—C5—H5B	109.6	O1—C10—H10B	109.5
C6—C5—H5B	109.6	H10A—C10—H10B	109.5
H5A—C5—H5B	108.1	O1—C10—H10C	109.5
C9—C4—C3	112.07 (13)	H10A—C10—H10C	109.5
C9—C4—C5	110.57 (13)	H10B—C10—H10C	109.5
C3—C4—C5	109.96 (13)	O2—C11—H11A	109.5
C9—C4—H4	108	O2—C11—H11B	109.5
C3—C4—H4	108	H11A—C11—H11B	109.5
C5—C4—H4	108	O2—C11—H11C	109.5
C9—O91—H91	109.5	H11A—C11—H11C	109.5
N1—C6—C8	109.41 (13)	H11B—C11—H11C	109.5
			
C6—N1—C2—C7	−179.88 (13)	O2—C8—C6—N1	159.66 (13)
C6—N1—C2—C3	−56.88 (17)	O4—C8—C6—C5	102.51 (18)
N1—C2—C7—O3	21.3 (2)	O2—C8—C6—C5	−75.56 (17)
C3—C2—C7—O3	−103.81 (18)	C4—C5—C6—N1	−55.54 (18)
N1—C2—C7—O1	−160.97 (13)	C4—C5—C6—C8	−178.22 (13)
C3—C2—C7—O1	73.94 (16)	C9—C4—C3—C2	−177.93 (13)
O3—C7—O1—C10	2.2 (2)	C5—C4—C3—C2	−54.53 (17)
C2—C7—O1—C10	−175.62 (14)	N1—C2—C3—C4	57.03 (17)
C6—C5—C4—C9	178.34 (13)	C7—C2—C3—C4	−179.89 (13)
C6—C5—C4—C3	54.06 (17)	O4—C8—O2—C11	0.9 (2)
C2—N1—C6—C8	179.01 (13)	C6—C8—O2—C11	178.96 (15)
C2—N1—C6—C5	55.96 (17)	C3—C4—C9—O91	−56.50 (19)
O4—C8—C6—N1	−22.3 (2)	C5—C4—C9—O91	−179.57 (13)

### Hydrogen-bond geometry (Å, °)

**Table:**

*D*—H···*A*	*D*—H	H···*A*	*D*···*A*	*D*—H···*A*
O91—H91···N1^i^	0.82	2.07	2.883 (2)	171
N1—H1···O4^ii^	0.98 (2)	2.19 (2)	3.144 (2)	165 (2)

Symmetry codes: (i) *x*, *y*+1, *z*; (ii) −*x*+2, −*y*, −*z*.
